# Pharmacotherapy of elderly patients in everyday anthroposophic medical practice: a prospective, multicenter observational study

**DOI:** 10.1186/1471-2318-10-48

**Published:** 2010-07-21

**Authors:** Elke Jeschke, Thomas Ostermann, Manuela Tabali, Horst C Vollmar, Matthias Kröz, Angelina Bockelbrink, Claudia M Witt, Stefan N Willich, Harald Matthes

**Affiliations:** 1 Havelhoehe Research Institute, Kladower Damm 221, 14089 Berlin, Germany; 2 Center of Integrative Medicine, University of Witten/Herdecke, Gerhard-Kienle-Weg 4, 58313 Herdecke, Germany; 3 Institute for General Practice and Family Medicine, University of Witten/Herdecke, Alfred-Herrhausen-Str. 50, 58448 Witten, Germany; 4 Deutsches Zentrum für Neurodegenerative Erkrankungen e.V. (DZNE) in cooperation with Witten/Herdecke University, Stockumer Str. 10, 58453 Witten, Germany; 5 Institute for Social Medicine, Epidemiology and Health Economics, Charité University Medical Centre, 10117 Berlin, Germany

## Abstract

**Background:**

Pharmacotherapy in the older adult is a complex field involving several different medical professionals. The evidence base for pharmacotherapy in elderly patients in primary care relies on only a few clinical trials, thus documentation must be improved, particularly in the field of complementary and alternative medicine (CAM) like phytotherapy, homoeopathy, and anthroposophic medicine. This study describes diagnoses and therapies observed in elderly patients treated with anthroposophic medicine in usual care.

**Methods:**

Twenty-nine primary care physicians in Germany participated in this prospective, multicenter observational study on prescribing patterns. Prescriptions and diagnoses were reported for each consecutive patient. Data were included if patients were at least 60 years of age. Multiple logistic regression analysis was used to determine factors associated with anthroposophic prescriptions.

**Results:**

In 2005, a total of 12 314 prescriptions for 3076 patients (68.1% female) were included. The most frequent diagnoses were hypertension (11.1%), breast cancer (3.5%), and heart failure (3.0%). In total, 30.5% of the prescriptions were classified as CAM remedies alone, 54.4% as conventional pharmaceuticals alone, and 15.1% as a combination of both. CAM remedies accounted for 41.7% of all medications prescribed (35.5% anthroposophic). The adjusted odds ratio (AOR) for receiving an anthroposophic remedy was significantly higher for the first consultation (AOR = 1.65; CI: 1.52-1.79), treatment by an internist (AOR = 1.49; CI: 1.40-1.58), female patients (AOR = 1.35; CI: 1.27-1.43), cancer (AOR = 4.54; CI: 4.12-4.99), arthropathies (AOR = 1.36; CI: 1.19-1.55), or dorsopathies (AOR = 1.34; CI: 1.16-1.55) and it decreased with patient age (AOR = 0.97; CI: 0.97-0.98). The likelihood of being prescribed an anthroposophic remedy was especially low for patients with hypertensive diseases (AOR = 0.36; CI: 0.32-0.39), diabetes mellitus (AOR = 0.17; CI: 0.14-0.22), or metabolic disorders (AOR = 0.17; CI: 0.13-0.22).

**Conclusion:**

The present study is the first to provide a systematic overview of everyday anthroposophic medical practice in primary care for elderly patients. Practitioners of anthroposophic medicine prescribe both conventional and complementary treatments. Our study may facilitate further CAM-research on indications of, for example, dementia or adverse drug reactions in the elderly.

## Background

With average life expectancy increasing and birth rates declining, the proportion of elderly people expands in almost every developed country. Due to this demographic shift, in Germany the proportion of elderly people is expected to rise from 20 to 34% within the next decades. Particularly, the proportion of people aged 80 years or older will rise from 5% today to 14% in 2060 [1].

Bearing this development in mind, pharmacotherapy in an aging society is a challenge that will be faced in upcoming years. Increasing age will go hand-in-hand with the prevalence of many diseases and this will, therefore, lead to higher prescription drug consumption. According to data from German health insurance companies, in 2005 the proportion of drug users among the elderly was 91%. Additionally, 55% of patients over age 75 produced 90% of drug-related costs [2].

While there is a broad field of research that analyzes pharmacological treatments in the middle aged population, there is a substantial knowledge gap in the study of pharmacotherapy in elderly patients [3]. This is mainly due to the fact that available data on pharmacotherapy in the elderly is widespread and older people are often excluded from trials. Even in cases where there is clinical data on the effects of pharmacology in elderly patients, prescribing patterns in primary care are often unknown, which is peculiar considering the elderly are the main users of pharmacotherapy.

One aspect that has more or less been neglected in health services research is the prescription of complementary drugs to elderly patients in primary care. The use of complementary and alternative medicine (CAM) among elderly persons is of worldwide significance and of increasing interest [4-7]. Some evidence has also shown that CAM is used for elderly patients with special conditions, like cancer [8,9], hypertension [10], or mental disorders [11]. This is often the result of self-medication but it is also prescribed by physicians. For instance, 14.5% of women with cancer consulted an alternative practitioner in Australia within a span of 12 months [9]. In the elderly, concurrent use of CAM products and conventional medicines was found to be common [12].

There are only a few hints as to when, how, and in what amount physicians, particularly those related to CAM, prescribe complementary medicine drugs to elderly patients. To fill this knowledge gap, the EvaMed project was launched by physicians specializing in anthroposophic medicine (AM) [13]. AM is a medical system that was founded in the 1920s by Rudolf Steiner and Ita Wegman [14]. Apart from physical therapies like rhythmical embrocations [15], therapeutic eurythmy [16], or creative art therapies [17], AM offers a broad variety of pharmacological therapies. AM, however, is not an isolated system; it regards itself as an extension of conventional treatment, requiring the physician to combine both conventional and anthroposophic treatments to an individualized and personalized treatment regimen to stimulate the capacity of the patient for self-healing.

Anthroposophic remedies are based on preparations of mineral, botanical, or zoological origins as well as chemically defined substances that are either undiluted or based on the homoeopathic principle of high dilution. In the field of oncology, for example, anthroposophic remedies based on mistletoe extracts are the most important drugs in Germany. According to a survey by Münstedt et al., every third GP (35%) prefers mistletoe extracts for the treatment of cancer patients [18]. Anthroposophical pharmacotherapy also provides combined preparations. Cardiodoron^®^, for example, a composition of extracts from *Primula officinalis *and *Onopordon acanthium *blossoms combined with extracts from the herb *Hyoscyamus niger*, is frequently prescribed for cardio-respiratory coordination, such as for patients with orthostatic symptoms [19].

Implemented in the German Drug Law, AM together with homeopathy and phytotherapy are summarized as "special therapeutic directions". Note that pharmaceutical ingredients like *Hypericum perforatum *(St. John's wort) by means of their manufacturing may occur in all three special therapeutic directions. On the drug level, however, the classification is distinct; every CAM-related drug therapy according to the pharmaceutical preparation is assigned only to one of these directions.

To offer insight into the field of CAM-related pharmacotherapy in elderly patients, this analysis aims to investigate the prescribing patterns for this group of patients.

## Methods

In total, 29 primary care physicians in Germany participated in this prospective, multicenter observational study. All were members of the EvaMed Pharmacovigilance Network, which since 2004 has aimed to evaluate complementary remedies in usual care with regard to prescribing patterns, efficacy, and safety [13,20]. Physicians were recruited through the German National Association of Anthroposophic Physicians (*Gesellschaft Antroposophischer Ärzte in Deutschland*; GAÄD). A total of 362 physicians were contacted and informed about the EvaMed Network by standard mail and, in the event of no response, four weeks later by telephone. For a physician to be eligible to participate in the study, his or her medical practice had to meet a number of technical requirements, including the presence of a special computerized patient documentation system (DocExpert, DocConcept, TurboMed, Duria, AdamedPlus, Medistar), a local area network (LAN) connection, and Microsoft Windows and Internet Explorer (i.e. as client software). A total of 38 physicians (10.5%) fulfilled the technical requirements, gave informed consent, and agreed to participate in the EvaMed Network. Of these physicians, the 9 who specialized in paediatrics were excluded from the study. Each of the remaining 29 physicians had practised for at least five years in primary care in addition to completing training in anthroposophic medicine.

The study period lasted from January through December 2005. Data were included in the analysis if patients were at least 60 years old and had received drug treatment at least once during the study period.

During the study, participating physicians continued to follow their routine documentation procedures, recording all treatment relevant diagnoses and all prescriptions for each consecutive patient using their existing computerized patient documentation system. These data were exported to the QuaDoSta postgreSQL-database system hosted in each practice [21]. After completing each export, participating physicians used a browser-based interface to match individual diagnoses with the corresponding drugs or remedies that had been prescribed. After receiving the data sets, study investigators checked the data for completeness (e.g. matching diagnoses with remedies). If necessary, the study center phoned the physicians and asked them to supply any missing data.

Diagnoses were coded according to the 10th revision of the International Classification of Diseases (ICD-10). Prescribed drugs were documented using the German National Drug Code (*German: Pharmazentralnummer*; PZN). Medications were classified as anthroposophic, homoeopathic, phytotherapeutic, or conventional according to the German ABDA database (ABDA = German acronym for the Federal Confederation of German Pharmacist Associations), which contains a broad range of data on all currently available medicinal drugs and substances. 'Anthroposophic', 'homoeopathic', and 'phytotherapeutic' were defined according to the regulations of the German Drug Law and are referred to below as 'CAM remedies'. Overall medication regimens were classified as consisting of CAM remedies alone, conventional pharmaceuticals alone, or a combination of both.

The present study is based on secondary data provided by physicians. As such, the recommendations for good practice in secondary data analysis (e.g. anonymization of data on prescriptions and diagnoses) developed by the German Working Group on the Collection and Use of Secondary Data [22] were applied in full. In addition, the study was approved by the responsible data security official.

Statistical analysis was performed with SPSS 16.0 for Windows. Descriptive analysis was used to determine prescription rates. Means and standard deviations (SD) were calculated for continuous data. In cases where data were not normally distributed, medians and interquartile ranges (IQR) were reported. The two-tailed chi-square test was used to analyze differences in prescription rates and the Cochran-Armitage test was used as a measure of age-related trends in prescription rates. The Kruskal-Wallis test was used to analyze differences in medians of prescriptions and prescribed medications among groups. The significance level was set at α = 0.05. Subgroup analyses were performed for patient gender, age (60-74 years; 75-79 years, 80 years or older), and diagnosis (ICD-10 chapters), as well as for drug type, medication regimen, physician specialization, and consultation type (first vs. follow up).

As a precursor to multivariate analysis, univariate analysis with bonferroni correction was carried out to determine factors associated with the prescription of anthroposophic remedies. We included consultation type (first vs. follow up), physician specialization, patient age and sex, ICD10-diagnoses groups, number of diagnoses, and number of prescribed remedies at admission as relevant variables in our analysis. Associations were analysed in the complete study population and across age strata.

A multiple logistic regression model was then conducted using a stepwise backward selection based on the likelihood ratio statistics. Adjusted odds ratios (AOR) and 95% confidence intervals (CI) were calculated. The dependent variable was the prescribing of any anthroposophic remedy. Significant results of univariate analysis that were independly associated with prescribing anthroposophic remedies were included as independent variables in the logistic regression model. Patient age was introduced in the model as a continuous, centered variable. Multicollinearity and interactions between independent predictors were investigated. The Hosmer-Lemeshow goodness-of-fit test was used to assess how well the chosen model fits the data (χ^2 ^= 87.16; *df *= 8; *P *< 0.001).

## Results

### Physicians

Of the 29 participating physicians, 21 (72%) were GPs and 8 (28%) were specialists (including 4 (14%) internists). The participating physicians did not differ significantly from the overall population of physicians certified in anthroposophic treatment in Germany in 2005 (n = 362) in terms of mean age (49.1 ± 6.1 years vs. 47.5 ± 6.1 years; *P *= 0.175) or gender (58.1% vs. 62.2% males; *P *= 0.662) and they were only slightly younger and consisted of a similar percentage of women compared to all office-based physicians in Germany (mean 52.0 years; 61.2% men) [23].

### Patients and prescriptions

During the one-year study period in 2005, a total of 12 314 prescriptions for 3076 patients aged 60 years or older (68.1% female) were included. Altogether, 2103 (68.4%; 66.4% female) of the patients were 60-74 years, 391 (12.7%; 62.9% female) were 75-79 years, and 582 (18.9%; 77.7% female) were 80 years or older. The median age was 69 (IQR [65, 77]). Each patient had a median of 2 (IQR [1,5]) prescriptions per year.

### Diagnoses

Table 1 provides a detailed overview of treatment diagnoses according to patient age and gender. In total, 23.5% of the reported diagnoses were diseases of the circulatory system, 13.9% were cancer, 10.6% were diseases of the musculoskeletal system and connective tissue, 8.1% were endocrine, nutritional and metabolic diseases, 7.7% were diseases of the nervous system, and 6.8% were mental and behavioural disorders. Other diseases range far behind. The most frequent single diagnoses were essential hypertension (11.1%), breast cancer (3.5%), heart failure (3.0%), depression (2.6%), Parkinson's disease (2.5%), and chronic ischemic heart disease (2.3%).

**Table 1:** Most frequent diagnoses according to age group and gender

Diagnosis [ICD-10]	Total [%]	Female [%]	Male [%]
**60-74 years (n = 2127)**			

Hypertensive diseases [I10-I15]	25.1	23.3	28.6
Malignant neoplasms, stated or presumed to be primary, of specified sites, except of lymphoid, haematopoietic, and related tissue [C00-C75]	19.0	20.8	15.5
Mood [affective] disorders [F30-F39]	11.9	13.0	7.2
Dorsopathies [M40-M54]	10.7	10.1	12.0
Arthropathies [M00-M25]	9.1	10.1	7.1
Episodic and paroxysmal disorders* [G40-G47]	8.3	9.0	6.8
Other forms of heart disease [I30-I52]	8.3	8.1	8.6
General symptoms and signs [R50-R69]	8.2	8.5	7.7
Chronic lower respiratory diseases [J40-J47]	8.0	8.1	7.9
Diabetes mellitus [E10-E14]	6.3	4.4	10.0

**75-79 years (n = 396)**			

Hypertensive diseases [I10-I15]	33.6	31.9	36.6
Other forms of heart disease [I30-I52]	16.4	15.0	19.0
Malignant neoplasms, stated or presumed to be primary, of specified sites, except of lymphoid, haematopoietic, and related tissue [C00-C75]	12.6	8.3	20.4
Mood [affective] disorders [F30-F39]	10.9	12.6	7.7
Ischaemic heart diseases [I20-I25]	10.6	10.2	11.3
Diabetes mellitus [E10-E14]	10.1	12.2	6.3
Dorsopathies [M40-M54]	9.8	11.8	6.3
Arthropathies [M00-M25]	9.8	10.6	8.5
Chronic lower respiratory diseases [J40-J47]	9.3	7.5	12.7
Organic, including symptomatic, mental disorders** [F00-F09]	6.8	6.7	7.0

**80 years or older (n = 553)**			

Hypertensive diseases [I10-I15]	39.4	39.6	38.8
Other forms of heart disease [I30-I52]	32.9	33.3	22.3
Ischaemic heart diseases [I20-I25]	16.8	16.7	17.4
Arthropathies [M00-M25]	15.7	17.6	9.1
Dorsopathies [M40-M54]	12.8	12.3	14.9
Episodic and paroxysmal disorders* [G40-G47]	12.3	13.0	9.9
Organic, including symptomatic, mental disorders** [F00-F09]	11.6	11.1	13.2
Osteopathies and chondropathies [M80-M94]	9.9	12.3	1.7
Chronic lower respiratory diseases [J40-J47]	9.8	8.8	13.2
Mood [affective] disorders [F30-F39]	9.4	9.5	9.1
Malignant neoplasms, stated or presumed to be primary, of specified sites, except of lymphoid, haematopoietic, and related tissue [C00-C75]	9.4	8.6	12.4

* including sleep disorders, ** including dementia

Every patient had an average of 3.33 ± 3.10 different treatment diagnoses per year (median: 2; IQR [1,4]). In total, 48.7% of patients had more than 2 diagnoses (3 or 4 diagnoses: 22.4%, more than 4 diagnoses: 26.3%). The number of diagnoses increased with patient age (more than 2 diagnoses: 60-74 years: 44.2%; 75-79 years: 50.6; > 80 years: 61.7%) and was higher for women than men (more than 2 diagnoses: 50.6% vs. 44.8%).

### Pharmacological therapy

**Figure 1:**
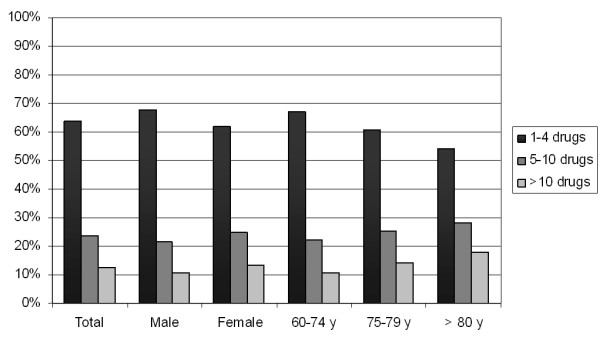
Number of prescribed drugs per patient and year according to gender and age group

Of the 28 961 medications prescribed, physicians recorded a median of 1 (IQR [1,2]) drug and remedy per patient and consultation. Every patient had a median of 4 (IQR [2,11]) medications per year. Figure 1 provides a detailed overview of the number of prescribed medications per patient during the one year study period. 63.7% of the patients were prescribed 1-4 different drugs, 23.8% were prescribed 5-10 drugs, and 12.5% of patients were prescribed more than 10 drugs per year.

Table 2 gives a detailed overview of the medication regimens prescribed by the participating physicians. In total, 30.5% of the regimens could be classified as CAM remedies alone, 54.4% as conventional pharmaceuticals alone, and 15.1% as a combination of CAM remedies and conventional pharmaceuticals. There were significant differences in medication regimen according to patient age (*P *< 0.001) and gender (*P *< 0.001), physician specialization (*P *< 0.001), consultation type (*P *< 0.001), and for various diagnoses (*P *< 0.001). For example, the proportion of the therapy regimen 'only CAM treatment' decreased significantly with patient age (36.2%; 60-74 years, 23.2%; 75-79 years, and 21.7% over 80 years; *P *for trend < 0.001) and was higher in women than men (32.7% vs. 25.3%; *P *< 0.001).

**Table 2:** Therapy regimen according to consultation type, physician specialization, patient age, gender, and diagnosis

	Total	Only CAM treatment		CAM and conventional treatment combined		Only conventional treatment	
	**N**	**n**	**%**	**n**	**%**	**n**	**%**

**Total**	**12314**	**3758**	**30.5**	**1858**	**15.1**	**6698**	**54.4**

**Consultation type**							
First	1845	664	36.0	139	7.5	1042	56.5
Follow-up	10469	3094	29.6	1719	16.4	5656	54.0

**Physician specialization**							

GP	7956	2376	29.9	1161	14.6	4419	55.5
Internal medicine	2527	972	38.5	427	16.9	1128	44.6
Other	1831	410	22.4	270	14.7	1151	62.9

**Age (y)**							

60-74	7320	2648	36.2	1134	15.5	3538	48.3
75-79	1762	409	23.2	239	13.6	1114	63.2
> 80	3232	701	21.7	485	15.0	2046	63.3

**Sex**							

male	3567	901	25.3	513	14.4	2153	60.4
female	8747	2857	32.7	1345	15.4	4545	52.0

**Diagnosis [ICD-10]**							

Diseases of the circulatory system [I00-I99]	2835	563	19.9	424	15.0	1848	65.2

Neoplasms [C00-D48]	1380	856	62.0	137	9.9	387	28.0

Diseases of the musculoskeletal system and connective tissue [M00-M99]	1211	443	36.6	206	17.0	562	46.4

Endocrine, nutritional, and metabolic diseases [E00-E90]	1103	148	13.4	141	12.8	814	73.8

Diseases of the nervous system [G00-G99]	1058	189	17.9	119	11.2	750	70.9

Mental and behavioural disorders [F00-F99]	901	260	28.9	126	14.0	515	57.2

Diseases of the digestive system [K00-K93]	763	220	28.8	147	19.3	396	51.9

Diseases of the respiratory system [J00-J99]	745	308	41.3	155	20.8	282	37.9

Symptoms, signs, and abnormal clinical and laboratory findings [R00-R99]	695	215	30.9	133	19.1	347	49.9

Diseases of the genitourinary system [N00-N99]	386	124	32.1	50	13.0	212	54.9

Diseases of the skin and subcutaneous tissue [L00-L99]	376	136	36.2	67	17.8	173	46.0

Injury, poisoning, and certain other consequences of external causes [S00-T98]	263	80	30.4	35	13.3	148	56.3

Certain infectious and parasitic diseases [A00-B99]	260	71	27.3	62	23.8	127	48.8

Diseases of the eye and adnexa [H00-H59]	103	50	48.5	19	18.4	34	33.0

Other n < 100	235	95	40.4	37	15.7	103	43.8

Table 3 provides an overview of the most frequently prescribed conventional pharmaceuticals according to age group and gender. In total, conventional pharmaceuticals accounted for 58.3% of all medications prescribed. The most frequent substances were metoprolol (beta-blocker; 2.4% of all conventional pharmaceuticals), levothyroxine sodium (thyroid hormone; 1.9%), metamizole sodium (pyrazolone; 1.9%), acetylsaliyciclic acid (1.6%), enalapril (ACE inhibitor; 1.6%), dopa and dopa derivates (anti-Parkinson drug; 1.6%), and furosemide (diuretic; 1.4%). Antihypertensive medication (e.g. calcium channel blockers, diuretics, beta-blockers, angiotensin converting enzyme (ACE) inhibitors, angiotensin II receptor antagonists, alpha-1 blockers, and antiadrenergic agents) accounted for 20.0% (n = 3390) of all conventional drugs prescribed (11.7% of all drugs and remedies). The most frequently prescribed substances according to drug classes were as analgesics various opioids (50.9% of all analgesics) and pyrazolone (metamizole sodium; 33.7%), as psycholeptics benzodiazepine derivates (28.7% of all psycholeptics) and benzodiazepine related drugs (25.4%), as anti-dementia drugs anticholinesterases (18.0% of all anti-dementia drugs) and memantine (13.4%), as antidepressants amitriptyline (19.3% of all antidepressants) and doxepin (7.5%), as anti-Parkinson drugs dopa and dopa-derivates (45.6% of all anti-Parkinson drugs) and dopamine agonists (35.1%), and as lipid modifying agents simvastatine (61.6% of all lipid modifying agents).

**Table 3:** Most frequent conventional drugs according to age group and gender

Medication [ATC classification]	Total		Gender				Age (y)					
			
			Male		Female		60-74		75-79		> 80	
	N	%	n	%	n	%	n	%	n	%	n	%
**Total**	**16910**	**100.0**	**5661**	**100.0**	**11249**	**100.0**	**9303**	**100.0**	**2795**	**100.0**	**4812**	**100.0**

AGENTS ACTING ON THE RENIN-ANGIOTENSIN SYSTEM [C09]	1218	7.2	469	8.3	749	6.7	714	7.7	208	7.4	296	6.2

ANALGESICS [N02]	945	5.6	298	5.3	647	5.8	571	6.1	78	2.8	296	6.2

PSYCHOLEPTICS [N05]	823	4.9	281	5.0	541	4.8	400	4.3	144	5.2	279	5.8

DIURETICS [C03]	795	4.7	266	4.7	529	4.7	301	3.2	157	5.6	337	7.0

BETA BLOCKING AGENTS [C07]	740	4.4	275	4.9	465	4.1	453	4.9	116	4.2	171	3.6

PSYCHOANALEPTICS [N06]	711	4.2	242	4.3	469	4.2	329	3.5	135	4.8	247	5.1

CARDIAC THERAPY [C01]	648	3.8	205	3.6	358	3.2	215	2.3	116	4.1	317	6.6

ANTI-PARKINSON DRUGS [N04]	643	3.8	363	6.4	280	2.5	296	3.2	153	5.5	194	4.0

ANTITHROMBOTIC AGENTS [B01]	559	3.3	245	4.3	314	2.8	205	2.2	117	4.2	237	4.9

ANTIINFLAMMATORY AND ANTIRHEUMATIC PRODUCTS [M01]	552	3.3	148	2.6	404	3.6	316	3.4	78	2.8	158	3.3

DRUGS FOR ACID RELATED DISORDERS [A02]	506	3.0	150	2.6	356	3.2	300	3.2	57	2.0	149	3.1

CALCIUM CHANNEL BLOCKERS [C08]	489	2.9	191	3.4	298	2.6	208	2.2	125	4.5	156	3.2

THYROID THERAPY [H03]	440	2.6	63	1.1	377	3.4	319	3.4	61	2.2	60	1.2

DRUGS FOR OBSTRUCTIVE AIRWAY DISEASES [R03]	438	2.6	171	3.0	267	2.4	226	2.4	100	3.6	112	2.3

DRUGS USED IN DIABETES [A10]	426	2.5	167	3.0	259	2.3	274	2.9	76	2.7	76	1.6

MINERAL SUPPLEMENTS [A12]	422	2.5	79	1.4	343	3.0	254	2.7	44	1.6	124	2.6

ANTIBIOTICS [J01]	366	2.2	115	2.0	251	2.2	205	2.2	64	2.3	97	2.0

LIPID MODIFYING AGENTS [C10]	333	2.0	141	2.5	192	1.7	237	2.5	54	1.9	42	0.9

Other n < 300	5856	34.6	1792	31.7	4150	36.9	3480	37.4	912	32.6	1464	30.4

CAM remedies accounted for 41.7% of all medications prescribed (35.5% anthroposophic, 3.3% homeopathic, 2.9% phytotherapeutic). The proportion of CAM remedies was significantly higher for women than for men (33.2% vs. 46.1%; *P *< 0.001). Although the total number of prescribed CAM remedies remained stable, the proportion of CAM remedies decreased with patient age while conventional drugs were prescribed more often (48.1% 60-74 years, 32.1% 75-79 years, and 30.1% over 80 years; *P *for trend < 0.001). The phytopharmaceutical *ginkgo biloba *was the most frequently prescribed anti-dementia drug overall (68.6% of all anti-dementia drugs).

Of those 124 patients who received antidementiva, 92 were given *ginkgo biloba *alone (74.2%) and one patient was co-prescribed *ginkgo biloba *with anticholinesterases (0.8%).

*Hypericum perforatum *served as a phytopharmaceutical, homeopathic or anthroposophic remedy often prescribed for depression (45.5% of all antidepressants). Of those 184 patients who received antidepressants, 69 (37.5%) were prescribed *Hypericum perforatum *alone, while 4 patients were co-prescribed *Hypericum perforatum *with amitriptyline (2.2% of all depressive patients), and no patient was co-prescribed *Hypericum perforatum *with doxepin.

Altogether, 10 271 prescriptions for a total of 976 different anthroposophic remedies were prescribed during the study. Various mistletoe preparations (e.g. Abnobaviscum abietis^®^, Abnobaviscum mali^®^, Helixor P^®^, Iscador P^®^) accounted for 21.7% (n = 2233) of all anthroposophic remedies prescribed (7.7% of all drugs and remedies). In total, mistletoe was prescribed for 81.6% (413/506) of all patients with cancer. Other more frequent anthroposophic remedies for particular diagnoses were Cardiodoron^® ^and Aurum/Belladonna comp.^® ^for essential hypertension, Helleborus niger e planta tota^® ^for cancer, Oleum Strophanthi forte^® ^for heart failure, Solum Oil^® ^for dorsopathies^®^, and hypericum and various Aurum peparations for depression.

The proportion of prescribed anthroposophic medication significantly decreased with patient age (41.4% 60-74 years, 26.7% 75-79 years, and 25.2% over 80 years; *P *for trend < 0.001) and was higher for female than male patients (37.7% vs. 30.5%, *P *< 0.001). After bonferroni correction in univariate analysis, consultation type, physician specialization, and the following diagnoses were also found to be significant: hypertensive diseases, cancer, arthropathies, chronic lower respiratory diseases, diabetes mellitus, episodic and paroxysmal disorders, ischemic heart disease, extrapyramidal and movement disorders, dorsopathies, metabolic disorders, and disorders of the thyroid gland. Diagnoses with a proportion of anthroposophic medication over 60% were neoplasms (71.3%), as well as diseases of the eye and adnexa (68.6%), and less than 15% endocrine, nutritional, and metabolic disorders (14.7%). For diseases of the circulatory system, the most frequent disorders, only 24.4% of the drugs and remedies prescribed were anthropsophic medication.

**Figure 2:**
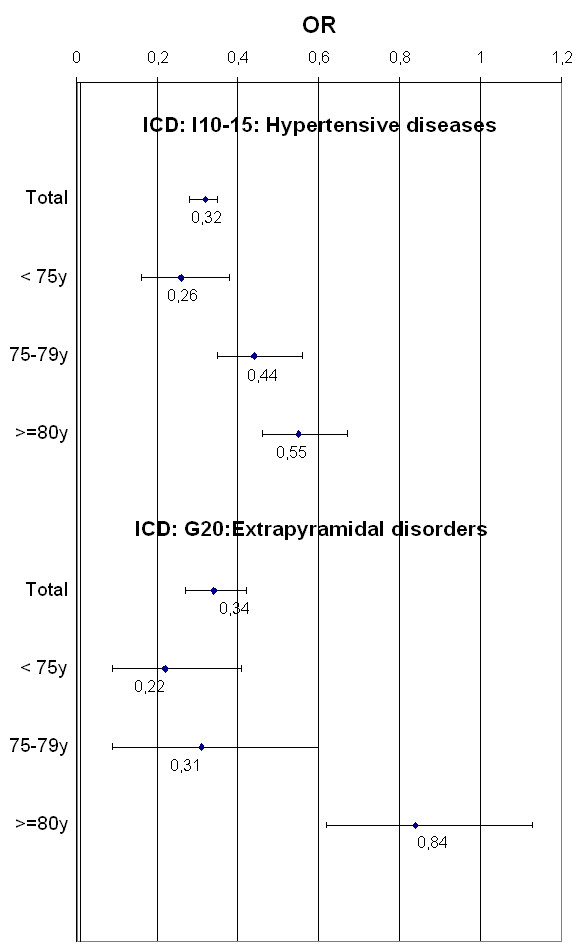
Odds ratios for the use of anthroposophic remedies for hypertensive disease and extrapyramidal and movement disorders stratified by age groups

Table 4 shows the adjusted odds ratio (AOR) for the most frequent diagnoses and other factors associated with being prescribed an anthroposophic remedy. Patient age and gender, as well as physician specialization, consultation type, and diagnoses, had an impact on the choice of remedy prescribed. The AOR for receiving an anthroposophic remedy was significantly greater than 1 for patients having their first consultation (AOR = 1.65; CI: 1.52-1.79), treatment by an internist (AOR = 1.49; CI: 1.40-1.58), female patients (AOR = 1.35; CI: 1.27-1.43), cancer (AOR = 4.54; CI: 4.12-4.99), arthropathies (AOR = 1.36; CI: 1.19-1.55), or dorsopathies (AOR = 1.34; CI: 1.16-1.55). The likelihood of being prescribed an anthroposophic remedy was especially low for patients with hypertensive diseases (AOR = 0.36; CI: 0.32-0.39), diabetes mellitus (AOR = 0.17; CI: 0.14-0.22), or metabolic disorders (AOR = 0.17; CI: 0.13-0.22). The interactions between hypertensive disease and age, as well as extrapyramidal and movement disorders and age, were found to be statistically significant (see Figure 2 for the odds ratios in the different age strata) and were included in the model. In the case of hypertensive disease and extrapyramidal and movement disorders, the likelihood of being prescribed an anthroposophic remedy increased with age.

**Table 4:** Multivariate logistic regression: factors associated with anthroposophic remedies

Factor	AOR (95% CI)
**Consultation type**	
Follow-up	1
First	1.65 (1.52 - 1.79)*

**Physician specialization**	
GP	1
Internal medicine	1.49 (1.40 - 1.58)*
Other	0.76 (0.70 - 0.84)*

Age (y)**	0.97 (0.97 - 0.98)*

**Sex**	
male	1
female	1.35 (1.27 - 1.43)*

**Diagnosis (groups)**	
Hypertensive diseases [I10-I15]	0.36 (0.32 - 0.39)*
Malignant neoplasms, stated or presumed to be primary, of specified sites, except of lymphoid, haematopoietic, and related tissue [C00-C75]	4.54 (4.12 - 4.99)*
Malignant neoplasms of ill-defined, secondary, and unspecified sites [C76-C80]	2.90 (2.54 - 3.30)*
Arthropathies [M00-M25]	1.36 (1.19 - 1.55)*
Chronic lower respiratory diseases [J40-J47]	0.59 (0.50 - 0.69)*
Diabetes mellitus [E10-E14]	0.17 (0.14 - 0.22)*
Episodic and paroxysmal disorders [G40-G47]	0.53 (0.45 - 0.63)*
Ischaemic heart diseases [I20-I25]	0.71 (0.60 - 0.83)*
Extrapyramidal and movement disorders [G20-G26]	0.46 (0.37 - 0.58)*
Dorsopathies [M40-M54]	1.34 (1.16 - 1.55)*
Metabolic disorders [E70-E90]	0.17 (0.13 - 0.22)*
Disorders of thyroid gland [E00-E07]	0.54 (0.45 - 0.64)*

**Interaction terms**	
Hypertensive diseases [I10-I15] * age	1.03 (1.02 - 1.04)*
Extrapyramidal and movement disorders [G20-G26] * age	1.04 (1.02 - 1.07)*

* OR significant different from 1; ** continuous centered variable

## Discussion

### Diagnoses

In accordance with the findings of Cheung et al., the most common conditions were chronic diseases [24]. Most of the patients in our cohort were treated for hypertension, cancer, depression, dorsopathies, and arthropathies. Hypertension, ischemic heart disease, and other forms of heart disease (i.e. heart failure) were the primary reasons patients 80 years and older visited a physician in our study. These diseases are also the main reasons patients aged 80 years or older are treated in conventional settings in Germany [25]. Compared to elderly patients treated by conventional physicians, the patients in this present study showed a lower proportion of diseases associated with a modern lifestyle, such as disorders of lipoprotein metabolism and diabetes mellitus [25,26]. This might be attributed to a higher educational level among patients treated by anthroposophic physicians, resulting in a more health-conscious lifestyle. As Unkelbach et al. has shown, patients of conventional and anthroposophic physicians in Germany are comparable in many aspects (e.g. age, proportion suffering from a chronic disease), but anthroposophic patients are more often female, a higher proportion of patients have a university degree, and a lower proportion are smokers or are overweight [27]. Other studies found CAM users tended to be younger and more educated and fewer are hypertensive or report, for example, arthritis, depression/anxiety [4,28], or pain [29].

Due to the fact that AM is well known for pharmacotherapeutic options, like mistletoe-preparations for adjuvant treatment of cancer, it is not surprising that our study counted for more patients with cancer in the present study cohort [30-32]. This also applies to depression because visiting an anthroposophic physician has shown specific benefits in terms of quality of life for patients suffering from depression [33]. However, this does not directly yield a higher prescription rate of AM drugs. Particularly in the field of depression, our physicians also follow the trend of prescribing herbal remedies, like St. John's wort, which are not directly linked to AM.

A study from Canada has shown that as patients age, multimorbidity plays an increasingly important role in primary care with a prevalence of 60% among people aged 55 to 74 [34]. According to van den Akker et al., male patients aged 80 and over had a mean of 3.25 diagnoses per year, whereas female patients had a mean of 3.57 [35]. Although our cohort was slightly younger (aged 60 and older), we found a comparable value of 3.33 diagnoses. Unsurprisingly and in accordance with other findings, the percentage of patients with comorbidity increased with older age and was higher for female patients [35].

### Pharmacological therapy

As already stated, we found an increment in medication directly related to the number of diagnoses. According to Laux et al., this interrelationship is mainly influenced by age and, to a lesser extend, by gender [36]. In the Slone survey, which evaluated the pattern of medication use in the ambulatory adult population of the United States, 23% of patients aged 65 years and over took 5 or more drugs, whereas 12% took more than 10 drugs within one week [37]. In contrast to our study, they also included OTC-medications in their calculations. Another study, which evaluated characteristics of outpatient prescriptions for the elderly in Taiwan, with an average age of 78 years, found that 84% of the observed population took 5 or more drugs per year [38]. These findings strongly differ from our results; the number of prescribed different drugs per patient and year was greater than 5 for only 36% of the patients and only 13% took more than 10 drugs per year. Although some factors, like the occurrence of polypharmacy, were more likely in female patients and in older patients [37,39], one has to be aware that a comparison of results has to take into account the different healthcare settings and policies in which the studies were conducted.

In the field of anthroposophic medicine, pharmacological treatment is based on an integrative concept that combines conventional and CAM approaches depending on the disease and it is carried out on a patient-by-patient basis. Regardless of diagnosis, CAM remedies alone were prescribed in 31% of cases, conventional pharmaceuticals alone in 54% of cases, and a combination of CAM remedies and conventional pharmaceuticals in 15% of cases. The likelihood of receiving CAM remedies alone was highest for patients with neoplasms (62% of all prescriptions for this group of patients) and lowest for patients with endocrine, nutritional, and metabolic disorders (13%). It is interesting to note that CAM medications were mainly prescribed for patients with cancer and dementia, whereas the majority of patients with cardiovascular and metabolic disorders were prescribed conventional medications. This is in accordance with a previous analysis [13]; however, there is no direct explanation for this trend. One hypothesis is that no additional treatment is used for diseases that can be controlled with conventional treatment, whereas in terms of cancer, for example, patients and physicians more often seek an additional treatment.

Of course, these observations do not infer that conditions like cancer are not conventionally treated (e.g. by chemotherapy or surgery). In such cases, AM is prescribed in addition to conventional treatment sometimes over a longer period of time [40]. Mistletoe in this respect was the most frequently prescribed anthroposophic remedy. These findings partially confirm those reported in a study by Hamre et al., who also found high prescription rates for anthroposophic remedies in patients with cancer [41]. According to a recently published review, supportive mistletoe therapy seems safe and particularly beneficial for the quality of life of adult patients with solid tumours [30]. However, anthroposophic remedies were also prescribed quite frequently for dorsopathies alone or as a substitute for conventional remedies, showing an improvement in health-related quality of life and long-term stabilization [42]. For elderly patients with depression, various Hypericum and Aurum preparations were frequently prescribed. In accordance with the results of an earlier study, anthroposophic remedies, such Cardiodoron^® ^and Aurum/Belladonna comp.^®^, were frequently prescribed for hypertension, mainly as adjuncts to classic antihypertensive therapy most likely as a way to stimulate and harmonize the rhythmic system and to assist the body in regulating blood pressure with its own resources [19,43].

Patient gender was associated with significant differences in prescription rates in the present study. This is in accordance with the results of several studies, which found that gender had an influence on the rate at which different medications were prescribed for adults and the female sex was a predictor of CAM use in the elderly [44-46]. The present study showed that older patients are less likely to receive an anthroposophic remedy. Compared to GPs, internists prescribed anthroposophic remedies more often to patients and issued a significantly larger number of conventional and CAM medications per patient. This may be attributed to a tendency among patients to seek help from specialists in cases of severe disease.

With regard to conventional drugs, only minor differences were observed in prescription rates in the present study compared to those in the German Drug Prescription Report (*Arzneimittelverordnungsreport*) from 2006, an annual publication listing, among other data, of the proportion of prescriptions most frequently issued in Germany [47]. Indeed, in the present study, patients were most likely to receive medication for hypertension, followed by analgesics, psycholeptics, psychoanaleptics, and anti-Parkinson drugs.

The successful treatment of hypertension in elderly patients seems to be the most important drug therapy that reduces the agglomeration of morbidity at the end of life as shown in the HYVET-trial (Hypertension in the Very Elderly Trial) [48,49]. Our findings suggest that such patients are mainly treated with ACE-antagonists and diuretics. While the amount of diuretics increased with age, the amount of beta-blockers and ACE-inhibitors decreased. In accordance with other studies, in younger populations we were able to show that male patients were more likely to be prescribed an ACE-antagonist [50,51]. Altogether, hypertension was treated in accordance with the German guidelines. We give a more detailed analysis of hypertensive treatment elsewhere [43].

Another important condition is dementia. In our setting, *Ginkgo biloba *is the most frequently prescribed complementary drug for dementia. The appraisal of evidence for the treatment of dementia is inconsistent [52]. One of the latest studies showed that ginkgo is an effective adjuvant treatment option for elderly patients with mild cognitive impairment [53], whereas the German Institute for Quality and Efficiency in Health Care (IQWIG) found only a benefit for the 240 mg preparation and criticized the heterogeneity of the study results [54]. However, German GPs vote ginkgo preparations for dementia as equally effective as anticholinesterases and memantine [55].

With regard to mental disorders in non-institutionalized elderly people, St. John's wort (*Hypericum perforatum*) is the most often prescribed antidepressant followed by amitriptylin and doxepin [56]. We were able to confirm these results in our primary care network. Also, other age related diseases like Parkinson's disease showed similar prescribing patterns found in other studies with a strong increase in male patients and patients aged 70 years and older [57-59].

### Implications for practice and policy

The provision of CAM in primary care still needs further education and information from primary care health professionals about its potential and limitations, particularly when combining CAM remedies with conventional drugs. The chronological dimension of pharmacotherapy in the treatment of elderly patients and the occurrence of critical combinations is a topic of notably high interest to health services research. Although we did not find a significant amount of such events, our network offers possibilities to further research this topic. In addition, the associations between AM prescribing and factors like age, sex, consultation type, and diagnoses do not, to our knowledge, have a correlation in other prescription studies in primary care and should also be investigated more closely. Finally, the use of over-the-counter (OTC) medications by elderly patients is a field of interest. With only limited data on the use of CAM and OTC drugs in the elderly, physicians should record their use systematically, which would make them available for research in settings like this one.

### Limitations

The present study has some limitations. Firstly, although physician prescribing data were subjected to an internal review as described above, coding inaccuracies cannot be ruled out entirely. In contrast to other methods of conducting epidemiological surveys for prescribing patterns in primary care, QuaDoSta is linked to the existing computerized patient documentation systems of physicians and can be incorporated fully into their daily routines, avoiding typical biases, such as missing data or double entries. Although such an approach may not have an impact on external validity, it can increase the internal validity of data [22]. According to Himmel (2006), we nevertheless can assume that relevant data like patient age, gender, and prescriptions are sufficiently documented by the physicians and that it is possible to use routine data for identification and classification of therapeutic actions, as well as for quality assurance in ambulatory patient care [60]. Secondly, additional data on specific diagnoses, such as information on disease severity and duration or the impact of these factors on the observed prescribing patterns, were unavailable. Thirdly, we were unable to obtain data on the subsequent medication use of patients who decided to switch physicians; similarly, no data were available for cases in which physicians provided patients with a referral for treatment by a specialist or hospital. Up to now, we also were not effectual in implementing our technology in a comparison group of conventional physicians. Finally, data on patient self-medication with CAM remedies or OTC drugs were unavailable. This may explain why only 36% of patients in this study took more than 5 medications while the numbers reported in the literature differ significantly.

## Conclusion

This study of a large sample of elderly people undergoing anthroposophic primary care in Germany is the first to provide a consecutive and systematic overview of pharmacotherapy in everyday conditions. During the 12-month study period, anthroposophic remedies accounted for 36% of all medications prescribed. Regardless of diagnosis, the likelihood of receiving an anthroposophic remedy was higher for female patients, patients having their first consultation, and patients treated by an internist. Cancer, arthropathies, and dorsopathies were the diagnoses for which anthroposophic remedies were prescribed most frequently. However, the likelihood of being prescribed an anthroposophic remedy decreased with patient age and was especially low for patients with hypertensive diseases, diabetes mellitus, or metabolic disorders.

The findings show that physicians who practice anthroposophic medicine prescribe both conventional and complementary medicine, thus taking an integrative approach. Our study may facilitate further CAM-research on indications of, for example, dementia or adverse drug reactions in the elderly.

## Competing interests

The authors declare that they have no competing interests.

## Authors' contributions

EJ participated in the design of the study, acquisition of data, performed the statistical analysis, and drafted the manuscript. TO made substantial contribution to the interpretation of data and statistical analysis. MT participated in the acquisition of data. HCV, AB, MK, MT, and CMW helped with the interpretation of the data, drafting, and critical revision of the manuscript. HM conceived of the study and participated in its design and coordination. MK, CMW, SNW, and HM have given final approval for the version to be published. All authors read and approved the final manuscript.

## Pre-publication history

The pre-publication history for this paper can be accessed here:

http://www.biomedcentral.com/1471-2318/10/48/prepub

## References

[B1] Statistisches Bundesamt Bevölkerung Deutschlands bis 2060. 12. koordinierte Bevölkerungsvorausberechnung Wiesbaden 2009

[B2] Glaeske G Jahnsen K GEK-Arzneimittel-Report 2007: Auswertungsergebnisse der GEK-Arzneimitteldaten aus den Jahren 2005 - 2006 2007 Sankt Augustin: Asgard-Verl. Hippe: GEK-Gmünder Ersatzkasse vol. GEK-Edition Band 59

[B3] Van Spall HG Toren A Kiss A Fowler RA Eligibility criteria of randomized controlled trials published in high-impact general medical journals: a systematic sampling review JAMA 2007 297 11 1233 1240 10.1001/jama.297.11.1233 17374817

[B4] Astin JA Pelletier KR Marie A Haskell WL Complementary and alternative medicine use among elderly persons: one-year analysis of a Blue Shield Medicare supplement J Gerontol A Biol Sci Med Sci 2000 55 1 M4 9 10719766 10.1093/gerona/55.1.m4

[B5] Najm W Reinsch S Hoehler F Tobis J Use of complementary and alternative medicine among the ethnic elderly Altern Ther Health Med 2003 9 3 50 57 12776475

[B6] Zeilmann CA Dole EJ Skipper BJ McCabe M Low Dog T Rhyne RL Use of herbal medicine by elderly Hispanic and non-Hispanic white patients Pharmacotherapy 2003 23 4 526 532 10.1592/phco.23.4.526.32117 12680482

[B7] Zhang AL Xue CC Lin V Story DF Complementary and alternative medicine use by older Australians Ann N Y Acad Sci 2007 1114 204 215 10.1196/annals.1396.032 17986583

[B8] Hana G Bar-Sela G Zhana D Mashiach T Robinson E The use of complementary and alternative therapies by cancer patients in northern Israel Isr Med Assoc J 2005 7 4 243 247 15847205

[B9] Sibbritt D Adams J Easthope G Young A Complementary and alternative medicine (CAM) use among elderly Australian women who have cancer Support Care Cancer 2003 11 8 548 550 10.1007/s00520-003-0490-4 12856200

[B10] Bell RA Suerken CK Grzywacz JG Lang W Quandt SA Arcury TA CAM use among older adults age 65 or older with hypertension in the United States: general use and disease treatment J Altern Complement Med 2006 12 9 903 909 10.1089/acm.2006.12.903 17109582

[B11] Grzywacz JG Suerken CK Quandt SA Bell RA Lang W Arcury TA Older adults' use of complementary and alternative medicine for mental health: findings from the 2002 National Health Interview Survey J Altern Complement Med 2006 12 5 467 473 10.1089/acm.2006.12.467 16813511

[B12] Elmer GW Lafferty WE Tyree PT Lind BK Potential interactions between complementary/alternative products and conventional medicines in a Medicare population Ann Pharmacother 2007 41 10 1617 1624 10.1345/aph.1K221 17785609 PMC2864004

[B13] Jeschke E Ostermann T Tabali M Bockelbrink A Witt CM Willich SN Matthes H Diagnostic profiles and prescribing patterns in everyday anthroposophic medical practice--a prospective multi-centre study Forsch Komplementmed 2009 16 5 325 333 10.1159/000235239 19887811

[B14] Steiner R Wegmann I Extending practical medicine: fundamental principles based on the science of the spirit GA 27 2000 Bristol: Rudolf Steiner Press 1 144

[B15] Ostermann T Blaser G Bertram M Michalsen A Matthiessen PF Kraft K Effects of rhythmic embrocation therapy with solum oil in chronic pain patients: a prospective observational study Clin J Pain 2008 24 3 237 243 10.1097/AJP.0b013e3181602143 18287830

[B16] Bussing A Ostermann T Majorek M Matthiessen PF Eurythmy Therapy in clinical studies: a systematic literature review BMC Complement Altern Med 2008 8 8 10.1186/1472-6882-8-8 18377647 PMC2322948

[B17] Hamre HJ Witt CM Glockmann A Ziegler R Willich SN Kiene H Anthroposophic art therapy in chronic disease: a four-year prospective cohort study Explore (NY) 2007 3 4 365 371 17681256 10.1016/j.explore.2007.04.008

[B18] Munstedt K Entezami A Kullmer U [Oncologic mistletoe therapy: physicians' use and estimation of efficiency] Dtsch Med Wochenschr 2000 125 41 1222 1226 10.1055/s-2000-7727 11076260

[B19] Cysarz D Heckmann C Kummell HC [The effects of Cardiodoron on cardio-respiratory coordination--a literature review] Forsch Komplementarmed Klass Naturheilkd 2002 9 5 292 297 10.1159/000067523 12417806

[B20] Jeschke E Ostermann T Luke C Tabali M Kroz M Bockelbrink A Witt CM Willich SN Matthes H Remedies containing Asteraceae extracts: a prospective observational study of prescribing patterns and adverse drug reactions in German primary care Drug Saf 2009 32 8 691 706 10.2165/00002018-200932080-00007 19591533

[B21] Jeschke E Luke C Ostermann T Tabali M Hubner J Matthes H [Prescribing practices in the treatment of upper respiratory tract infections in anthroposophic medicine] Forsch Komplementmed 2007 14 4 207 215 10.1159/000104171 17848797

[B22] AGENS Good practice of secondary data analysis, first revision] Gesundheitswesen 2008 70 1 54 60 10.1055/s-2007-1022529 18273764

[B23] Kassenärztliche Bundesvereinigung: Grunddaten zur vertragsärztlichen Versorgung 2008 http://www.zi-berlin.de/morbilitaetsanalyse/downloads/ADT-Panel-version3.pdf

[B24] Cheung CK Wyman JF Halcon LL Use of complementary and alternative therapies in community-dwelling older adults J Altern Complement Med 2007 13 9 997 1006 10.1089/acm.2007.0527 18047447

[B25] Grobe TG Dörning H Schwartz FW GEK-Report ambulant-ärztliche Versorgung 2008 2007 St. Augustin: Asgard-Verlag GEK-Edition Band 67 edn

[B26] Kerek-Bodden H Koch H Brenner G Flatten G [Diagnostic spectrum and treatment requirements of general practice clients. Results of the ADT Panel of the Central Institute of National Health Insurance Management] Z Arztl Fortbild Qualitatssich 2000 94 1 21 30 10721161

[B27] Unkelbach R Abholz HH [Differences between patients of conventional and anthroposophic family physicians] Forsch Komplementmed 2006 13 6 349 355 10.1159/000096224 17200609

[B28] Cherniack EP Senzel RS Pan CX Correlates of use of alternative medicine by the elderly in an urban population J Altern Complement Med 2001 7 3 277 280 10.1089/107555301300328160 11439850

[B29] Dello Buono M Urciuoli O Marietta P Padoani W De Leo D Alternative medicine in a sample of 655 community-dwelling elderly J Psychosom Res 2001 50 3 147 154 10.1016/S0022-3999(00)00223-3 11316507

[B30] Melzer J Iten F Hostanska K Saller R Efficacy and safety of mistletoe preparations (Viscum album) for patients with cancer diseases. A systematic review Forsch Komplementmed 2009 16 4 217 226 10.1159/000226249 19729932

[B31] Carlsson M Arman M Backman M Flatters U Hatschek T Hamrin E A Five-year Follow-up of Quality of Life in Women with Breast Cancer in Anthroposophic and Conventional Care Evid Based Complement Alternat Med 2006 3 4 523 531 10.1093/ecam/nel042 17173117 PMC1697754

[B32] Legnani W Mistletoe in conventional oncological practice: exemplary cases Integr Cancer Ther 2008 7 3 162 171 10.1177/1534735408319894 18956494

[B33] Hamre HJ Witt CM Glockmann A Ziegler R Willich SN Kiene H Anthroposophic medical therapy in chronic disease: a four-year prospective cohort study BMC Complement Altern Med 2007 7 10 10.1186/1472-6882-7-10 17451595 PMC1876246

[B34] Fortin M Lapointe L Hudon C Vanasse A Multimorbidity is common to family practice: is it commonly researched? Can Fam Physician 2005 51 244 245 16926936 PMC1472978

[B35] van den Akker M Buntinx F Metsemakers JF Roos S Knottnerus JA Multimorbidity in general practice: prevalence, incidence, and determinants of co-occurring chronic and recurrent diseases J Clin Epidemiol 1998 51 5 367 375 10.1016/S0895-4356(97)00306-5 9619963

[B36] Laux G Kuehlein T Rosemann T Szecsenyi J Co- and multimorbidity patterns in primary care based on episodes of care: results from the German CONTENT project BMC Health Serv Res 2008 8 14 10.1186/1472-6963-8-14 18205916 PMC2244601

[B37] Kaufman DW Kelly JP Rosenberg L Anderson TE Mitchell AA Recent patterns of medication use in the ambulatory adult population of the United States: the Slone survey JAMA 2002 287 3 337 344 10.1001/jama.287.3.337 11790213

[B38] Chan DC Hao YT Wu SC Characteristics of outpatient prescriptions for frail Taiwanese elders with long-term care needs Pharmacoepidemiol Drug Saf 2009 18 4 327 334 10.1002/pds.1712 19180586

[B39] Gokce Kutsal Y Barak A Atalay A Baydar T Kucukoglu S Tuncer T Hizmetli S Dursun N Eyigor S Saridogan M Polypharmacy in the elderly: a multicenter study J Am Med Dir Assoc 2009 10 7 486 490 10.1016/j.jamda.2009.03.018 19716065

[B40] Friedel WE Matthes H Bock PR Zanker KS Systematic Evaluation of the Clinical Effects of Supportive Mistletoe Treatment within Chemo- and/or Radiotherapy Protocols and Long-Term Mistletoe Application in Nonmetastatic Colorectal Carcinoma: Multicenter, Controlled, Observational Cohort Study J Soc Integr Oncol 2009 7 4 137 145 19883529

[B41] Hamre HJ Kiene H Kienle GS Clinical research in anthroposophic medicine Altern Ther Health Med 2009 15 6 52 55 19943577

[B42] Hamre HJ Witt CM Glockmann A Wegscheider K Ziegler R Willich SN Kiene H Anthroposophic vs. conventional therapy for chronic low back pain: a prospective comparative study Eur J Med Res 2007 12 7 302 310 17933703

[B43] Jeschke E Ostermann T Vollmar HC Kroz M Bockelbrink A Witt CM Willich SN Matthes H Evaluation of prescribing patterns in a German network of CAM physicians for the treatment of patients with hypertension: a prospective observational study BMC Fam Pract 2009 10 1 78 10.1186/1471-2296-10-78 20003298 PMC2804584

[B44] Cherniack EP Ceron-Fuentes J Florez H Sandals L Rodriguez O Palacios JC Influence of race and ethnicity on alternative medicine as a self-treatment preference for common medical conditions in a population of multi-ethnic urban elderly Complement Ther Clin Pract 2008 14 2 116 123 10.1016/j.ctcp.2007.11.002 18396255

[B45] Loera JA Reyes-Ortiz C Kuo YF Predictors of complementary and alternative medicine use among older Mexican Americans Complement Ther Clin Pract 2007 13 4 224 231 10.1016/j.ctcp.2007.03.002 17950177 PMC2100426

[B46] Tanaka MJ Gryzlak BM Zimmerman MB Nisly NL Wallace RB Patterns of natural herb use by Asian and Pacific Islanders Ethn Health 2008 13 2 93 108 10.1080/13557850701830349 18425709

[B47] Anlauf M Schwabe U, Paffrath D Antihypertonika. Arzneiverordnungs-Report 2006. Aktuelle Daten, Kosten, Trends und Kommentare 2006 Springer Verlag 408 424

[B48] Tulner LR Kuper IM van Campen JP Koks CH Mac Gillavry MR Beijnen JH Brandjes DP Treatment of hypertension in an elderly outpatient population in the Netherlands Am J Geriatr Pharmacother 2009 7 4 204 209 10.1016/j.amjopharm.2009.08.002 19766952

[B49] Beckett NS Peters R Fletcher AE Staessen JA Liu L Dumitrascu D Stoyanovsky V Antikainen RL Nikitin Y Anderson C Treatment of hypertension in patients 80 years of age or older N Engl J Med 2008 358 18 1887 1898 10.1056/NEJMoa0801369 18378519

[B50] Gu Q Burt VL Paulose-Ram R Dillon CF Gender differences in hypertension treatment, drug utilization patterns, and blood pressure control among US adults with hypertension: data from the National Health and Nutrition Examination Survey 1999-2004 Am J Hypertens 2008 21 7 789 798 10.1038/ajh.2008.185 18451806

[B51] Gasse C Hense HW Stieber J Doring A Liese AD Heller G Keil U Factors associated with differences in antihypertensive drug treatment: results from the MONICA Augsburg Population Surveys 1989/90 and 1994/95 Soz Praventivmed 2002 47 2 128 142 10.1007/BF01318395 12134731

[B52] Birks J Grimley Evans J Ginkgo biloba for cognitive impairment and dementia Cochrane Database Syst Rev 2007 2 CD003120 17443523 10.1002/14651858.CD003120.pub2

[B53] Baurle P Suter A Wormstall H Safety and effectiveness of a traditional ginkgo fresh plant extract - results from a clinical trial Forsch Komplementmed 2009 16 3 156 161 10.1159/000213167 19657199

[B54] Ginkgohaltige Präparate bei Alzheimer Demenz http://www.iqwig.de/download/A05-19B_Abschlussbericht_Ginkgohaltige_Praeparate_bei_Alzheimer_Demenz.pdf

[B55] van den Bussche H Kaduszkiewicz H [Prescription patterns and effectiveness perception of anti-dementia drugs - A comparison between General Practitioners, Neurologists and Psychiatrists] Nervenheilkunde 2005 24 485 492

[B56] Hach I Rentsch A Krappweis J Kirch W [Psychopharmaceutical prescriptions to older people. A comparison between patients in aged- and nursing homes, outpatient treatment with nursing care and outpatients without nursing care] Z Gerontol Geriatr 2004 37 3 214 220 10.1007/s00391-004-0180-y 15224242

[B57] Trifiro G Savica R Morgante L Vanacore N Tari M Moretti S Galdo M Spina E Caputi AP Arcoraci V Prescribing pattern of anti-Parkinson drugs in Southern Italy: cross-sectional analysis in the years 2003-2005 Parkinsonism Relat Disord 2008 14 5 420 425 10.1016/j.parkreldis.2007.10.010 18316232

[B58] Trifiro G Sini G Sturkenboom MC Vanacore N Mazzaglia G Caputi AP Cricelli C Brignoli O Aguglia E Biggio G Samani F Prescribing pattern of antipsychotic drugs in the Italian general population 2000-2005: a focus on elderly with dementia Int Clin Psychopharmacol 2010 25 1 22 8 10.1097/YIC.0b013e3283334f08 19898244

[B59] Coca V Nink K Schröder H Schwabe U, Paffrath D Arneiverordnungen nach Alter und Geschlecht Arzneiverordnungs-Report 2007 Aktuelle Daten, Kosten, Trends und Kommentare 2007 Springer Verlag 919 1044

[B60] Himmel W Hummers-Pradier E Kochen MM [Health services research in general practice. A new approach] Bundesgesundheitsblatt Gesundheitsforschung Gesundheitsschutz 2006 49 2 151 159 10.1007/s00103-005-1215-2 16429308

